# The removal of uranium onto carbon-supported nanoscale zero-valent iron particles

**DOI:** 10.1007/s11051-014-2813-4

**Published:** 2014-12-23

**Authors:** Richard A. Crane, Thomas Scott

**Affiliations:** School of Physics, Interface Analysis Centre, University of Bristol, Bristol, UK

**Keywords:** Nanoparticles, Zero-valent iron, Carbon black, Uranium, Remediation

## Abstract

In the current work carbon-supported nanoscale zero-valent iron particles (CS nZVI), synthesised by the vacuum heat treatment of ferric citrate trihydrate absorbed onto carbon black, have been tested for the removal of uranium (U) from natural and synthetic waters. Two types of CS nZVI were tested, one vacuum annealed at 600 °C for 4 h and the other vacuum annealed at 700 °C for 4 h, with their U removal behaviour compared to nZVI synthesised via the reduction of ferrous iron using sodium borohydride. The batch systems were analysed over a 28-day reaction period during which the liquid and nanoparticulate solids were periodically analysed to determine chemical evolution of the solutions and particulates. Results demonstrate a well-defined difference between the two types of CS nZVI, with greater U removal exhibited by the nanomaterial synthesised at 700 °C. The mechanism has been attributed to the CS nZVI synthesised at 700 °C exhibiting (i) a greater proportion of surface oxide Fe^2+^ to Fe^3+^ (0.34 compared to 0.28); (ii) a greater conversion of ferric citrate trihydrate [2Fe(C_6_H_5_O_7_)·H_2_O] to Fe^0^; and (iii) a larger surface area (108.67 compared to 88.61 m^2^ g^−1^). Lower maximum U uptake was recorded for both types of CS nZVI in comparison with the borohydride-reduced nZVI. A lower decrease in solution Eh and DO was also recorded, indicating that less chemical reduction of U was achieved by the CS nZVI. Despite this, lower U desorption in the latter stages of the experiment (>7 days) was recorded for the CS nZVI synthesised at 700 °C, indicating that carbon black in the CS nZVI is likely to have contributed towards U sorption and retention. Overall, it can be stated that the borohydride-reduced nZVI were significantly more effective than CS nZVI for U removal over relatively short timescales (e.g. <48 h), however, they were more susceptible to U desorption over extended time periods.

## Introduction

In recent years, nanoscale zero-valent iron particles (nZVI) have been investigated as a new tool for waste water treatment (Zhang 2003). Compared to ZVI commonly used in permeable reactive barriers (particle diameter > 0.1 μm), (Cantrell et al. 1995) nZVI have a significantly greater surface area to volume ratio, and resultantly, considerably enhanced reactivity with regard to contaminants (Zhang 2003). Their colloidal size also makes their deployment flexible due to their conceptually high mobility through porous media and their potential for injection at almost any location and depth in terrestrial groundwater systems (Crane and Scott 2012; Crane and Noubactep 2012). An intrinsic technical challenge, however, associated with the in situ immobilisation of metal and metalloid contaminants onto nZVI is the prospect for contaminant remobilisation (Crane and Scott 2012; Crane et al. 2015; Shin et al. 2013). This behaviour was recently documented by Crane et al. (2011), where nZVI were tested for the removal of U from dissolved oxygen (DO) bearing groundwater also containing a high concentration (>500 mg L^−1^) of dissolved bicarbonate. The nZVI were determined as initially effective for the removal of U, with >98 % (<10 µg/L) removal achieved within 2 h of reaction. However, over extended time periods (>7 days), significant (>50 %) re-release was recorded. The mechanism was attributed to incomplete chemical reduction of surface-precipitated U (from soluble U^6+^ to insoluble U^4+^), allowing the re-release of U concurrent with the re-ingress of DO and the subsequent reformation of highly stable (nominally carbonate) aqueous U-complexes. Indeed it has been demonstrated for circumneutral pH solutions lacking complexing agents (i.e. uranyl solution with conservative background electrolyte) near-total chemical reduction of U^6+^ to UO_2_(s) upon the surface of nZVI can occur (Riba et al. 2008), whereas the chemical reduction of U^6+^ is suppressed in solutions containing carbonate (Yan et al. 2010; Crane et al. 2011; Crane and Scott. 2014). Indeed O’Loughlin et al. (2003) confirmed that U^6+^ can be readily chemically reduced to U^4+^ by mixed Fe^2+^/^3+^ (oxy)hydroxide, however, they can also be readily re-oxidised when in the presence of DO. Furthermore, Gu et al. (1998) determined that reductively precipitated U^4+^ on Fe^0^ can be readily desorbed by introducing a carbonated-rich solution or allowing DO to ingress into the solution. Overall, the studies have highlighted that the utility of nZVI for the in situ removal of U from contaminated groundwater is likely to be restricted to anaerobic conditions since it is otherwise quickly transformed to iron (hydr)oxides under aerobic conditions, with concurrent U release (Crane and Scott 2012; Crane and Scott 2013; Scott et al. 2011; Yan et al. 2014). To address this issue, the use of (meso)porous support materials which provide additional sorption sites to prevent U desorption is an emerging field of research. Indeed nanocomposite materials may provide novel capability and function for the removal of metal and metalloid species from waste waters, due to the unique dual benefit of chemical reducing capability, facilitated by the nZVI, and high capacity sorption, facilitated by the mesoporous substrate. For example, resin-supported nZVI has been demonstrated to remove CrO_4_^2−^ and Pb^2+^ from aqueous solutions, with removal rates compared to ordinary nZVI enhanced by 5- and 18-fold, respectively (Ponder et al. 2000). In addition, nZVI supported by zeolite (Li et al. 2007) and clay minerals (Üzüm et al. 2009; Sheng et al. 2014) have demonstrated improvements in nZVI adsorption capacity. Mesoporous carbon (MC) exhibits unique physical (pore size distribution and surface area) and chemical functionality (Rahman et al. 2011) and has therefore received considerable interest as a candidate support material for nZVI. Materials investigated to date include granular-activated carbon-supported nZVI (Choi et al. 2009), multi-walled carbon nanotube-supported nZVI (Lv et al. 2011) and graphene-supported nZVI (Jabeen et al. 2011), with significant improvements in aqueous contaminant adsorption/degradation capacity recorded. The efficacy of MC-nZVI for the removal of U, however, remains a relatively unexplored research field, despite MC being well demonstrated as highly effective for U removal (Abbasi and Streat 1994; Chen et al. 2007; Kumar et al. 2011; Mellah et al. 2006; Wang et al. 2012). For example, to date there exists one study investigating the removal of U^6+^ onto reduced graphene oxide-supported nZVI with results demonstrating simultaneous U^6+^ adsorption and chemical reduction, along with significantly higher U removal capacity than unmodified nZVI (Suna et al. 2014). The comparative efficacies of numerous other forms of MC-nZVI are yet to be investigated. Carbon black is a low cost and abundant waste product from the fossil fuel industry and has been demonstrated as a suitable substrate material for the carbothermal synthesis of carbon-supported nZVI (Hoch et al. 2008). Analogous to the production of Fe^0^ from iron ore, the method regards the carbothermal reduction (>500 °C) of Fe^2+^ adsorbed onto carbon black. In addition, despite the high temperatures (>500 °C) required for the formation of Fe^0^ in preference to its oxides, the reaction is endothermic, with only gaseous by-products (H_2_, CO, CO_2_, etc.), and therefore represents an easily scalable process.

Carbon black-supported nZVI (CS nZVI) are tested in the current work for the removal of U from mine water and synthetic uranyl solutions. The work has been established in order to determine the differential U uptake behaviour of CS nZVI compared to conventional borohydride-reduced nZVI in solutions containing DO.

## Materials and methods

### Chemicals

All chemicals (carbon black, iron sulphate (FeSO4·7H_2_O), ferric citrate trihydrate [2Fe(C_6_H_5_O_7_)·H_2_O], nitric acid (HNO_3_), sodium borohydride (NaBH_4_), sodium hydroxide (NaOH), uranyl acetate [UO_2_(CH_3_COO)_2_·2H_2_O] and solvents [ethanol, acetone)] used in this study were of analytical grade and all solutions were prepared using Milli-Q purified water (resistivity > 18.2 MΩ cm). Carbon black (acetylene, 100 % compressed, 99.9+ %), surface area ~75 m^2^/g, bulk density 170–230 g/L was purchased from Alfa Aesar (CAS 1333-86-4).

### Nanoparticle synthesis

Carbon-supported nZVI were synthesised following an adaptation of the method described by Hoch et al. (2008). 37.01 g of ferric citrate trihydrate [2Fe(C_6_H_5_O_7_)·H_2_O] and 5 g of carbon black were combined in 500 mL of Milli-Q water (resistivity 18.2 MΩ cm at 25 °C) and stirred overnight using a DOS-10L Skyline autoshaker at 90 RPM. The resultant nanoparticle slurry was then decanted into 10-mL centrifuge vials and centrifuged in a Hamilton Bell Vanguard V6500 desktop centrifuge at 6,500 RPM for 30 s. The supernatant was then discarded and the wet nanoparticle powder was transferred into a vacuum desiccator and dried for 48 h at 1 × 10^−3^ mbar. The resulting particulates were then separated into two separate 2-g batches and heated under vacuum (<1 × 10^−3^ mbar) at 600 and 700 °C, respectively, for 4 h with a heating and cooling ramp rate of approximately 10 °C min^−1^. Fe^0^ is considered to form via the following reactions (Eqs. 1, 2).

1 $$2 {\text{Fe}}\left( {{\text{C}}_{ 6} {\text{H}}_{ 5} {\text{O}}_{ 7} } \right) \cdot {\text{H}}_{ 2} {\text{O}}_{{({\text{s}})}} + {\text{ C}}_{{({\text{s}})}} \to {\text{ 2Fe}}^{0}_{{({\text{s}})}} + {\text{ 2C}}_{ 3} {\text{H}}_{ 2} {\text{O}}_{{({\text{s}})}} + {\text{ 6CO}}_{{ 2({\text{g}})}} + {\text{ CO}}_{{({\text{g}})}} + {\text{ 5H}}_{ 2} {\text{O}}_{{({\text{l}})}}$$

2 $${\text{Fe}}_{ 3} {\text{O}}_{{ 4({\text{s}})}} + {\text{ 2C}}_{{({\text{s}})}} \to {\text{ 3Fe}}^{0}_{{({\text{s}})}} + {\text{ 2CO}}_{{ 2({\text{g}})}}$$

The resultant nanopowders were then stored in sealed containers in a nitrogen-filled (BOC, 99.998 %) glovebox (Saffron Scientific, Alpha series) until required.

Borohydride-reduced nZVI were synthesised following the method described by Wang and Zhang (1997), using sodium borohydride to reduce ferrous iron to a metallic state. 7.65 g of FeSO_4_·7H_2_O was dissolved in 50 mL of Milli-Q water and then a 4 M NaOH solution was used to adjust the solution pH to 6.8. The addition of NaOH was performed slowly, dropwise, to avoid the formation of hydroxocarbonyl complexes. The salts were reduced to metallic nanoparticles by the addition of 3.0 g of NaBH_4_. The nanoparticle product was isolated through centrifugation (Hamilton Bell v6500 Vanguard centrifuge, 6,500 RPM for 2 min) and then sequentially washed with water, ethanol and acetone (20 mL of each). The nanoparticles were then dried in a vacuum desiccator for 48 h at 1 × 10^−3^ mbar and stored in sealed containers in a nitrogen-filled (BOC, 99.998 %) glovebox (Saffron Scientific, Alpha series) until required.

### The mine water effluent

The mine water used in the current study was taken from the Lişava uranium mine, Banat, Romania. The mine site is valley confined and bounded by limestone ridges which contribute significant concentrations of dissolved bicarbonate to ground and surface waters, a complexing agent that is recognised to considerably enhance U-mobility in natural water systems (Ragnarsdottir and Charlet (2000). The water is used for mining and is pumped from approximately 200 m below sea level, a depth significantly beneath the water table. It initially contains low concentrations of DO (<3 mg L^−1^); however, it quickly equilibrates with the atmosphere to reach oxygen concentrations more typical for that of vadose and/or surface waters (~10 mg L^−1^), changing its redox potential and associated transport properties in the process.

### Experimental procedure

Four 500-mL Schott Duran jars were filled with 400 mL of the U-bearing mine water effluent. A further four jars were filled with 400 mL of Milli-Q water with U at 0.5 mg L^−1^, NaNO_3_ was then added at 0.1 mM as a background electrolyte species and the pH was adjusted to 8.5 using 0.01 M NaOH. The addition of NaOH was performed slowly, dropwise, to avoid the formation of hydroxocarbonyl complexes. 0.1 g of each nanoparticle type (borohydride-reduced nZVI, CS nZVI synthesised at 600 °C and CS nZVI synthesised at 700 °C) were then each added to the mine water and the uranyl solutions. Prior to addition, the nanopowders were each suspended in 1 mL of ethanol (Sigma-Aldrich, ≥99.5 %) and dispersed by sonication for 60 s using a Fisher Scientific Ultrasonic cleaner. The one remaining mine water solution and uranyl-only solution were retained as nanoparticle-free controls. Systems were sampled at 0, 1, 2, 4, 24, 48 h, 7, 14, 21 and 28 days. Prior to sampling, the batch systems were gently agitated to ensure homogeneity, and pH and Eh measurements were taken using a Hanna Instruments meter (model HI 8424) with a combination of gel electrode pH probe and a platinum ORP electrode, respectively. DO measurements were also taken using a Jenway 970 DO_2_ metre. Aliquots of 10 mL were also taken from each batch system and centrifuged using a Hamilton Bell Vanguard V6500 desktop centrifuge at 6,500 rpm for 30 s to separate the liquid and solid phases. The liquid was decanted, filtered through a 0.22-μm cellulose acetate filter and then prepared for ICP-AES and ICP-MS.

### Sample analysis methods

#### Bet

In preparation for BET surface area analysis, samples were degassed under vacuum (1 × 10^−2^ mbar) for a 12-h period at a temperature of 75 °C. A known weight of the dried material was measured with a Quantachrome NOVA 1200 surface area analyser, using N_2_ as the adsorbent and following a 7-point BET method.

#### ICP-AES preparation and conditions

The liquid samples were prepared for ICP-AES analysis by a 10 times dilution in 1 % nitric acid (analytical quality concentrated HNO_3_ in Milli-Q water). Blanks and standards for analysis were also prepared in 1 % nitric acid, with Fe standards of 0.10, 0.25, 0.50, 1.00, 2.50, 5.0 and 10.0 mg L^−1^. A Jobin–Yvon Ultima ICP-AES (sequential spectrometer) fitted with a cyclone spray chamber and a Burgener Teflon Mira mist nebulizer was used. The Fe concentration was measured using the emission line at 259.94 nm.

#### ICP-MS preparation and conditions

Samples from each batch system were prepared for ICP-MS analysis by a 20 times dilution in 1 % nitric acid (analytical quality concentrated HNO_3_ in Milli-Q water). Blanks and U standards at 1.0, 2.0, 10, 20 and 50 μg L^−1^ were also prepared in 1 % nitric acid. An internal Bi standard of 10 μg L^−1^ was added to blanks, standards and samples. The ICP-MS instrument used was a VG Thermo Elemental PQ 3.

#### Transmission electron microscopy

TEM images were obtained with a JEOL JEM 1200 EX Mk 2 TEM, operating at 120 keV. Nanoparticle samples were mounted on 200 mesh holey carbon-coated copper grids.

#### X-ray diffraction

A Phillips Xpert Pro diffractometer with a Cu_Kα_ radiation source (*λ* = 1.5406 Å) was used for XRD analysis (generator voltage of 40 keV; tube current of 30 mA). XRD spectra were acquired between 2*θ* angles of 0–90°, with a step size of 0.02° and a 2-s dwell time.

#### X-ray photoelectron spectroscopy

A Thermo Fisher Scientific Escascope equipped with a dual anode X-ray source (Al_Kα_ 1,486.6 eV and Mg_Kα_ 1,253.6 eV) was used for XPS analysis. Samples were analysed at <5×10^−8^ mbar with Al_Kα_ radiation of 300 W (15 kV, 20 mA) power. High-resolution scans were acquired using a 30 eV pass energy and 300-ms dwell times. Following the acquisition of survey spectra over a wide binding energy range, the Fe 2p, C 1 s, O 1 s and U 4f spectral regions were then scanned at a higher energy resolution such that valence state determinations could be made for each element. Data analysis was carried out using Pisces software (Dayta Systems Ltd.) with binding energy values of the recorded lines referenced to the adventitious hydrocarbon C1s peak at 284.8 eV. In order to determine the relative proportions of Fe^2+^ and Fe^3+^ in the sample analysis volume, curve fitting of the recorded Fe 2p photoelectron peaks was performed following the method of Grosvenor et al. (2004). The Fe 2p profile was fitted using photoelectron peaks at 706.7, 709.1, 710.6 and 713.4 eV corresponding to Fe^0^, Fe_octahedral_^2+^, Fe_octahedral_^3+^ and Fe_tetrahedral_^3+^. These parameters were selected on the basis that the surface oxide was assumed to be a mixture of wüstite and magnetite, as the oxide Fe^2+^ is in the same coordination with the surrounding oxygen atoms in both forms of oxide.

## Results

### Preliminary characterisation of the U-bearing mine water

Prior to nanoparticle addition, the U-bearing mine water was characterised using ICP-AES (Fe, Mg, Cu and Mo), ICP-MS (U), volumetric titration (HCO_3_^−^, NO_3_^−^ and PO_4_^3−^) and gravimetry (SO_4_^2−^) with supplementary Eh, pH and DO measurements, Table 1. The analysis indicated that HCO_3_^−^, well-documented to form uranyl complexes of high thermodynamic stability (Ragnarsdottir and Charlet 2000) was the most common ligand species present, with a concentration of 624 ppm.

**Table 1:** Concentrations of notable chemical species present in the mine water, analysed by ICP-MS (U), ICP-AES (Fe, Mg, Cu and Mo), volumetric titration (HCO_3_^−^, NO^3−^ and PO_4_^3−^) and gravimetry (SO_4_^2−)^ along with the recorded Eh, pH and DO

Chemical species	Concentration (ppm)
Cations	
Cu	0.043
Fe	0.019
Mg	16.18
Mo	0.031
U	0.545
Anions	
HCO_3_^−^	624
NO_3_^−^	19
PO_4_^3−^	0.3
SO_4_^2−^	1.2
Solution conditions	
DO (ppm)	11.3
Eh (mV)	234
pH	8.43

### Preliminary characterisation of nanoparticles

Vacuum annealing temperatures of 600 and 700 °C were used in the current work for the formation of CS nZVI in order to form Fe^0^ in preference to its oxides (Hoch et al. 2008). BET surface area analysis recorded a lower surface area for iron citrate sorbed onto carbon black in comparison with the original carbon black material (from 71.05 to 41.07 m^2^ g^−1^), Table 2. This is ascribed to a decrease in the porosity of the carbon black due to the adsorption of iron citrate. An increase in the surface area of the CS nZVI was recorded, however, as a result of vacuum annealing; with 59.06 and 63.96 m^2^ g^−1^ recorded for nanoparticles annealed for 2 h at 600 and 700 °C, respectively, Table 2. A further increase in CS nZVI surface area was also recorded for the nanoparticles vacuum annealed for 4 h, with 88.61 and 108.67 m^2^ g^−1^ recorded for CS nZVI 600 and 700 °C, respectively. This behaviour is ascribed to the decomposition of the carbon black material and the concurrent formation of nZVI (Eqs. 1, 2.). In contrast, the CS nZVI vacuum annealed for 12 h exhibited a lower surface area, with 76.31 and 39.08 m^2^ g^−1^ recorded for CS nZVI 600 and 700 °C, respectively. This is attributed to the diffusion bonding of individual nanoparticles during vacuum annealing (Scott et al. 2010; Dickinson et al. 2010). Consequently, for the vacuum annealing timescales tested, a thermal treatment period of 4 h was found to produce a material of optimal surface area.

**Table 2:** Changes in CS nZVI surface area as a function of vacuum annealing temperature and timescale

Nanoparticle type	Surface area (m^2^ g^−1^)
Carbon black	71.05
Carbon-supported nano-Fe^0^ (before annealing)	41.07
Carbon-supported nano-Fe^0^ (2 h 600 °C)	59.06
Carbon-supported nano-Fe^0^ (2 h 700 °C)	63.96
Carbon-supported nano-Fe^0^ (4 h 600 °C)	88.61
Carbon-supported nano-Fe^0^ (4 h 700 °C)	108.67
Carbon-supported nano-Fe^0^ (12 h 600 °C)	76.31
Carbon-supported nano-Fe^0^ (12 h 700 °C)	39.08

**Fig. 1:**
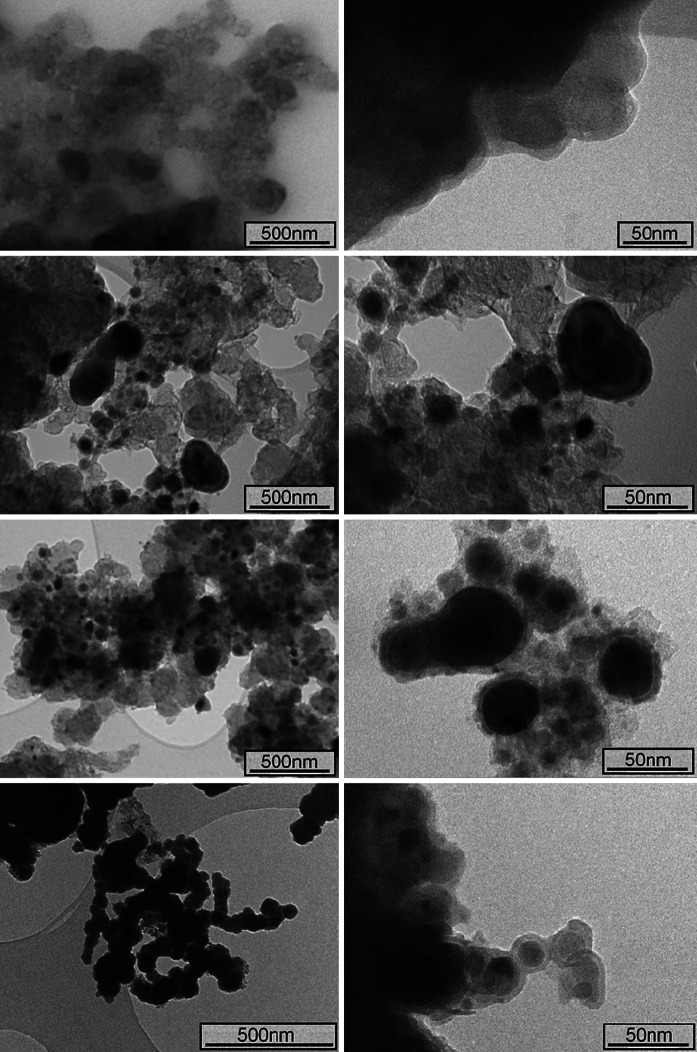
Transmission electron microscopy (TEM) images of: carbon-supported nZVI before vacuum annealing (*top*); carbon-supported nZVI after vacuum annealing for 4 h at 600 °C (*second from top*); carbon-supported nZVI after vacuum annealing for 4 h at 700 °C (*third from top*); and borohydride-reduced nZVI (*bottom*)

**Fig. 2:**
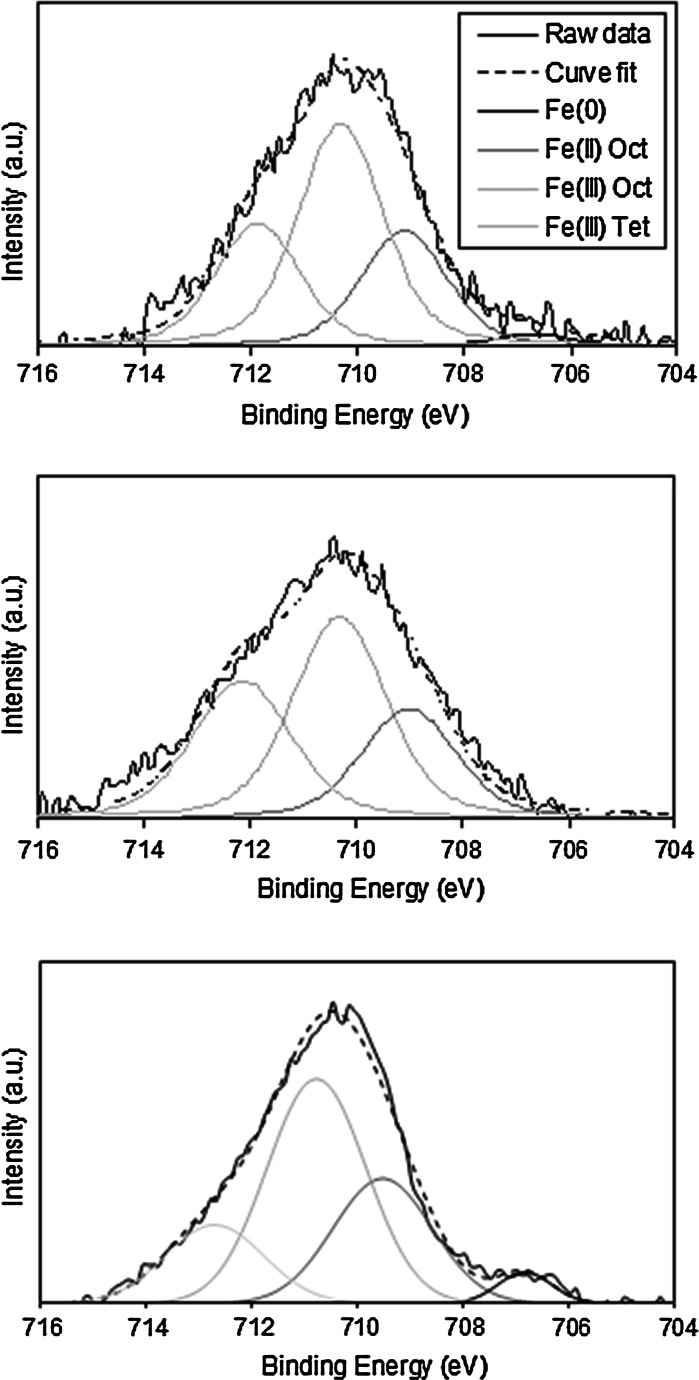
X-ray photoelectron spectroscopy (XPS) Fe 2p_3/2_ photoelectron profile recorded for carbon-supported nZVI that has been vacuum annealed at 700 °C (*top*) and 600 °C (*middle*), along with borohydride-reduced nZVI (*bottom*)

**Fig. 3:**
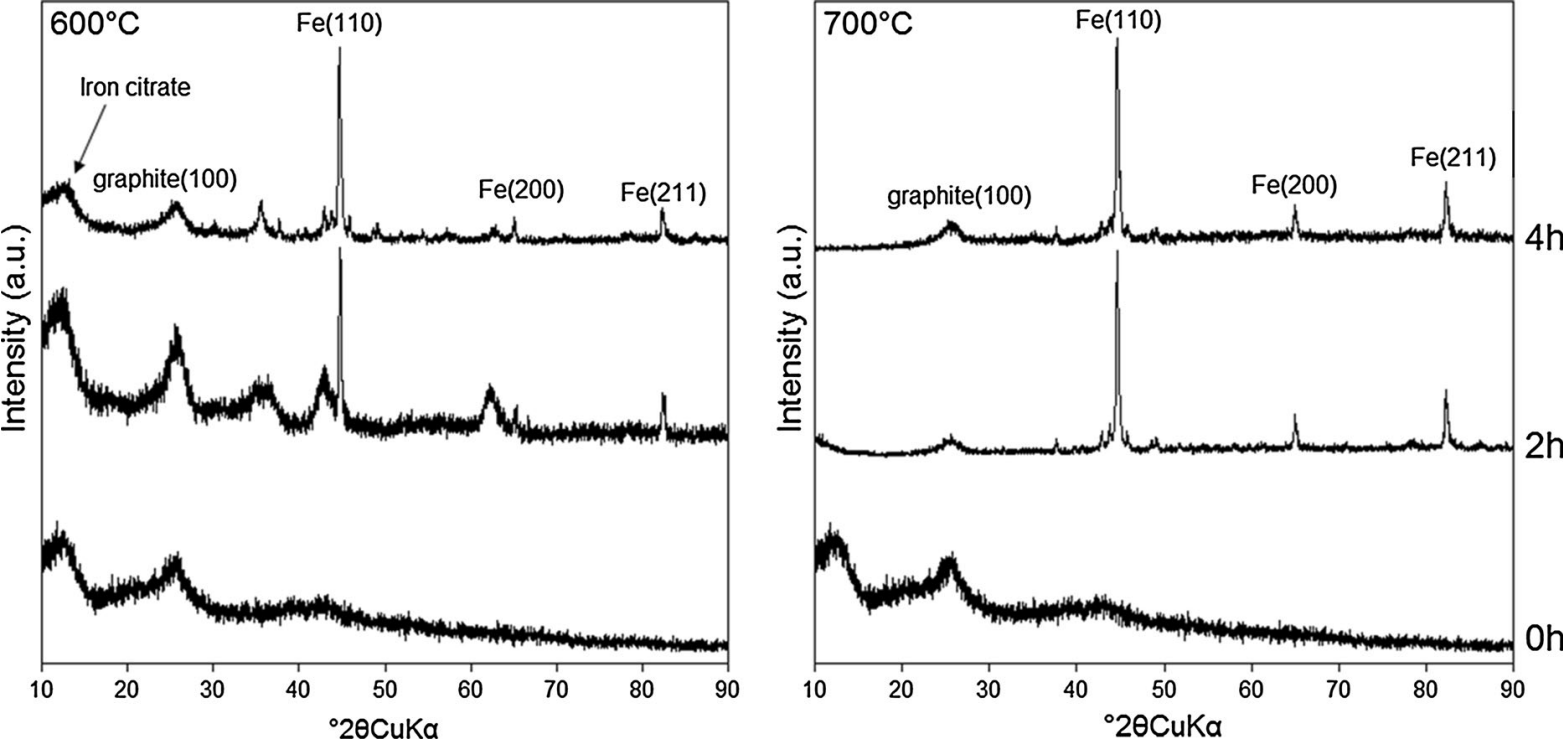
X-ray diffraction (XRD) spectra (for the range of 10–90° 2*θ*) recorded for the carbon-supported nZVI heated at 600 (LHS) and 700 °C (RHS) for 0 h (*bottom*), 2 h (*middle*) and 4 h (*top*)

Table 3 lists a summary of the experimental results regarding the bulk and surface properties of the CS nZVI and borohydride-reduced nZVI used for the U sorption experiments. No significant difference in size and/or morphology between the CS nZVI synthesised at 600 and 700 °C was recorded using TEM (Fig. 1), with both nanoparticle types determined as spherical and within an approximate size range of 20–150 nm. A greater concentration of a dense (dark coloured) crystalline material, however, was recorded for the CS nZVI formed at 700 °C. This is attributed to more complete conversion of ferric citrate trihydrate to Fe^0^ due to the higher temperature. XPS confirmed the presence of Fe^0^ within less than approximately 5 nm from the surface (the XPS analysis depth) for the CS nZVI formed at 700 °C, with a Fe^0^/(Fe^2+^ + Fe^3+^) ratio of 0.02 recorded (Fig. 2). A greater proportion of Fe^2+^ to Fe^3+^ was also recorded for the CS nZVI formed at 700 °C, with a ratio of 0.28 and 0.34 recorded for CS nZVI formed at 600 and 700 °C, respectively. XRD confirmed that, as a result of the higher temperature, a greater conversion of iron citrate trihydrate to Fe^0^ had occurred for the particles synthesised at 700 °C, Fig. 3.

**Table 3:** A summary of the experimental results regarding the bulk and surface properties of the CS nZVI and the borohydride-reduced nZVI

Parameter		nZVI	CS 600 °C	CS 700 °C
Particle size distribution (nm)	0–50	85 %	89 %	91 %
	51–100	8 %	6 %	5 %
	>100	7 %	5 %	4 %
Oxide thickness (nm)		3–4	3–5	3–5
BET surface area (m^2^/g)		19.03	88.61	108.67
Surface chemistry	(Fe(0)/Fe^2+^ + Fe^3+^)	0.05	n/a	0.02
	Fe^2+^/Fe^3+^	0.45	0.28	0.34

In comparison between the CS nZVI and the borohydride-reduced nZVI it can be noted that the latter nanomaterials were slightly in diameter (Fig. 1). In addition, the borohydride-reduced nZVI were more agglomerated than the CS nZVI materials, which are attributed to a decrease in the attraction forces (electrosteric and/or magnetic attraction) between individual nanoparticles caused by the presence of the carbon support. XRD analysis of the CS nZVI and the borohydride nZVI recorded three peaks centred at 44.6, 65.6 and 82.6° 2*θ*, implying the presence of Fe^0^, with lattice reflections Fe(110), Fe(200) and Fe(211), respectively. It can be noted that the peaks were much broader for the borohydride-reduced nZVI, indicating that the Fe^0^ is present in a more amorphous state than for the CS nZVI.

### Results from sorption experiments

#### Changes in DO/Eh/pH

The addition of nanoparticles to the water samples resulted in a rapid decrease in solution Eh for all systems studies; however, chemically reducing conditions was not achieved by either type of CS nZVI (Fig. 5). Similarly, a concurrent rapid decrease in DO was recorded, however, DO < 2 mg L^−1^ was not recorded for either CS nZVI type (Fig. 5). In contrast an Eh minima of −530 and −582 mV was recorded for the mine water and uranyl batch systems, respectively, containing borohydride-reduced nZVI. If we normalise the Eh change to surface area, the borohydride-reduced nZVI show significantly greater redox manipulation than the two CS nZVI types. For borohydride-reduced nZVI, values of 38.9 and 41.6 mV/m^2^ were recorded for the mine water and uranyl solution, respectively, whilst 2.1 and 1.9 mV/m^2^ were, respectively, recorded for the CS nZVI synthesised at 700 °C, and 2.0 and 1.7 mV/m^2^ were recorded for the CS nZVI synthesised at 600 °C.

**Fig. 4:**
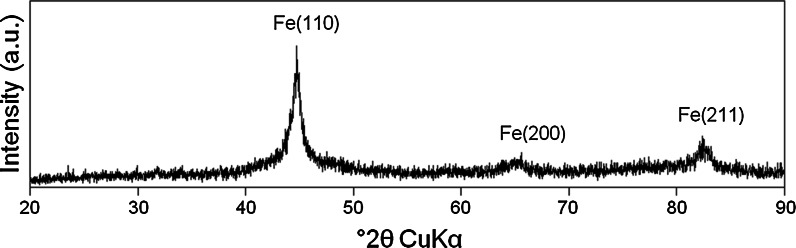
X-ray diffraction (XRD) spectra (for the range of 10–90° 2*θ*) recorded for the borohydride-reduced nZVI

**Fig. 5:**
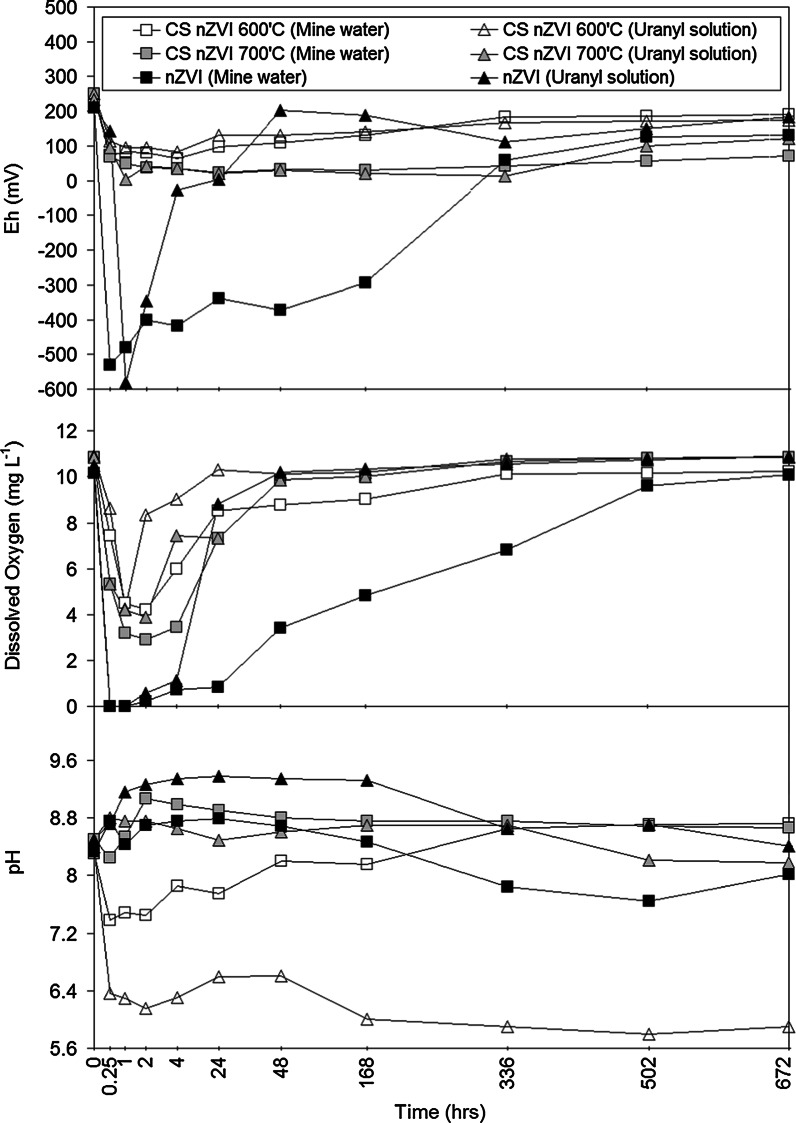
Changes in solution Eh, DO and pH as a function of reaction time (0–672 h) for the batch systems containing the different nZVI used in the current work. The control (nanoparticle free) batch system (not shown) recorded an Eh variation of <10 mV, a DO variation of < 0.2 mg L^−1^ and a pH variation of <0.05 from the starting solution

An accompanying increase in system pH was recorded for all batch systems (mine water and the uranyl solution) containing the CS nZVI that had formed at 700 °C and the borohydride-reduced nZVI (Figs. 4, 5). In contrast a decrease in pH was recorded in both batch systems containing the CS nZVI that had been formed at 600 °C, which is attributed to the dissolution of residual ferric citrate trihydrate [2Fe(C_6_H_5_O_7_)·H_2_O].

#### Changes in aqueous U and Fe concentration

**Fig. 6:**
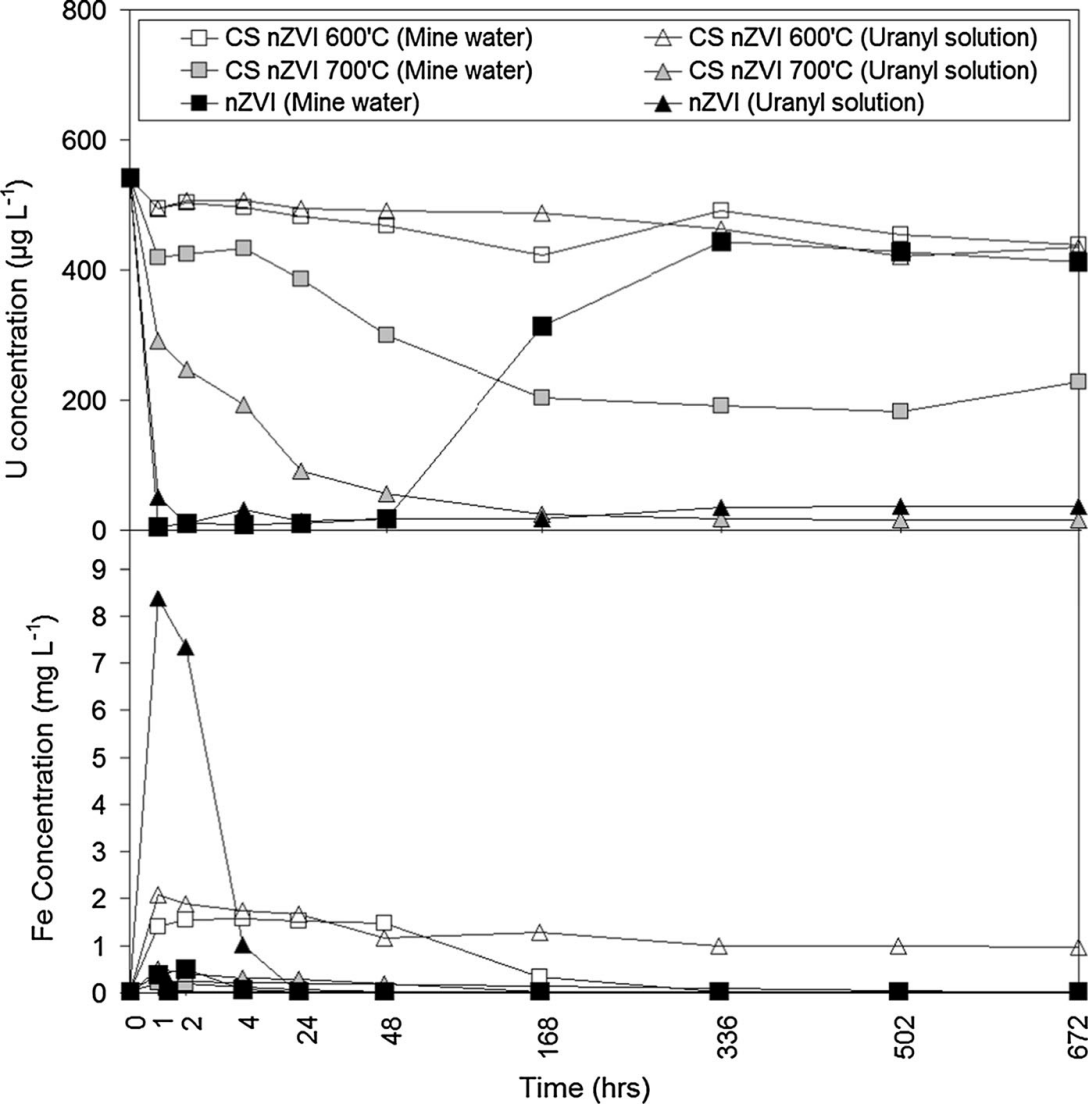
Changes in solution U and Fe concentration as a function of reaction time (0–672 h) for the batch systems containing the different nZVI used in the current work. The aqueous U control (nanoparticle free) batch system (not shown) recorded a variation of <1 mg L^−1^ from the starting solution concentrations

Analysis of aqueous samples extracted during period intervals using ICP-MS recorded limited removal of U in both batch systems (the mine water and the uranyl-only solution) treated using CS nZVI that had been formed at 600 °C (Fig. 6). Improved U removal was exhibited by the CS nZVI that had been formed at 700 °C, with near-total removal of U_(aq)_ recorded for the uranyl-only solution after 168 h (1 week) of reaction time and maximum removal of 67 % recorded for the mine water after 502 h (3 weeks) of reaction time.

Analysis of aqueous samples using ICP-AES extracted during period intervals recorded maximum Fe concentrations in all systems within the first 48 h of reaction (Fig. 6), which is attributed to the rapid corrosion of nanoparticulate surfaces. Greater Fe dissolution was recorded for batch systems (the groundwater and the uranyl-only solution) containing CS nZVI that had been formed at 600 °C, with a maximum of 1.56 and 2.08 mg L^−1^ recorded, respectively, compared to only 0.20 and 0.51 mg L^−1^ recorded for the CS nZVI that had been formed at 700 °C. This is attributed to the dissolution of residual ferric citrate trihydrate [2Fe(C_6_H_5_O_7_)·H_2_O] present in the CS nZVI that had been formed at 600 °C.

## Discussion

Analysis of the bulk structure, particle size and chemical composition of CS nZVI synthesised at 600 and 700 °C using BET surface area analysis, TEM, XPS and XRD recorded three key differences between the two nanoparticle types: (i) a greater proportion of surface oxide Fe^2+^ to Fe^3+^ recorded for the nanoparticles formed at 700 °C (0.28 compared to 0.34); (ii) a greater conversion of ferric citrate trihydrate [2Fe(C_6_H_5_O_7_)·H_2_O] to Fe^0^ recorded for the nanoparticles formed at 700 °C; and (iii) a larger surface area recorded for the nanoparticles formed at 700 °C (108.67 compared to 88.61 m^2^ g^−1^). It remains unclear which difference between would have contributed the most to the U uptake ability exhibited by CS nZVI formed at 700 °C. Regarding the first difference, the electrode potential of structural Fe^2+^ is in the range −0.65 to 0.34 V (Scott et al. 2010), which indicates that the oxidation of Fe^2+^ to Fe^3+^ is likely to have contributed to the chemical reduction of U^6+^. Indeed a number of studies have documented the reduction of U^6+^ to U^4+^ by structural (solid) Fe^2+^ (e.g. Fiedor et al. 1998). The relatively small difference in Fe^2+^ to Fe^3+^ ratios recorded for the CS nZVI synthesised at 600 and 700 °C (0.34 and 0.28, respectively), however, is likely to have caused this to have relatively negligible difference on the differential U uptake recorded. Regarding the second difference, it is likely that the greater conversion of 2Fe(C_6_H_5_O_7_)·H_2_O to Fe^0^ recorded for the CS nZVI synthesised at 700 °C would have enhanced U uptake via two closely coupled mechanisms: (i) the greater quantity of Fe^0^ would have allowed the material to exhibit greater chemical reducing capability and also a greater Fe^2+^ source for further U removal and chemical reduction (Charlet et al. 1998); and (ii) the lower quantity of residual 2Fe(C_6_H_5_O_7_)·H_2_O) would limit the aqueous formation of citric acid (C_6_H_8_O_7_) which is a well-known chelator of both Fe and U. Table 4 lists the stability constants for aqueous U^6+^ in the presence and absence of citric acid. It is shown that citric acid significantly raises the aqueous stability of uranyl.

**Table 4:** Stability constants (log K) of aqueous U^6+^ in the presence and absence of citric acid

Association reaction	Log K	Eq.
UO_2_^2+^ + H_2_O = UO_2_OH^+^ + H^+^	−5.2^a^	3
UO_2_^2+^ + 2H_2_O = UO_2_(OH)_2_ + 2H^+^	−12.0^a^	4
UO_2_^2+^ + 3H_2_O = UO_2_(OH)_3_^−^ + 3H^+^	−19.2^a^	5
UO_2_^2+^ + cit^3−^ = UO_2_cit^−^	8.74^a^	6
2UO_2_^2+^ + cit^3−^ = (UO_2_)_2_(cit)_2_^2−^	21.3^b^	7
H_3_cit = cit^3−^ + 3H^+^	−14.29^c^	8
H_2_cit = cit^3−^ + 2H^+^	−11.16^c^	9
Hcit^2−^ = cit^3−^ + H^+^	−6.39^c^	10

^a^Kantar et al. (2005), ^b^Smith and Martell (1993) and ^c^Field et al. (1974)

With regard to the third difference, it is well-documented that because aqueous contaminant sorption and chemical reduction on nZVI (and carbon-supported nZVI) is a surface-mediated process, the rate of contaminant uptake is therefore proportional to the surface area of the nanomaterial (Crane and Scott 2012). The higher surface area of the CS nZVI synthesised at 700 °C is therefore likely to have contributed to the increased capacity (or maxima) of the U uptake recorded. As discussed by Hoch et al. (2008), the differential surface area of the two materials is likely to have been due to an increase in the porosity of the carbon support due to carbonisation during the vacuum annealing process.

Lower maximum U removal was recorded for the CS nZVI synthesised at 700 °C than the borohydride-reduced nZVI. This is attributed in part due to the lower mass percentage of Fe^0^ in the former material. In addition, the kinetics of U uptake was recorded as slower for the CS nZVI, indicating that there was a lower density of surface sites for U sorption. A concurrent lower decrease in solution Eh and DO was recorded for both types of CS nZVI in comparison to the borohydride-reduced nZVI, indicating that the former exhibited a lower ability for U reduction. Despite this, lower U desorption in the latter stages of the experiment (>7 days) was recorded for the CS nZVI synthesised at 700 °C. The specific mechanism for this differential U uptake behaviour remains unclear, however, it is tentatively suggested that the presence of carbon black in the CS nZVI also contributed to the U removal, and may have limited the desorption of U. This was not unexpected given the well-documented association between carbon-based materials and aqueous uranium (Mossman et al. 1993; Nagy et al. 1998).

## Conclusions

In the current work, carbon-supported nanoscale zero-valent iron particles (CS nZVI) have been tested for the removal of U from natural and synthetic waters. Two types of CS nZVI were tested, one type that has been vacuum annealed at 600 °C for 4 h and the other type that has been vacuum annealed at 700 °C for 4 h, with their aqueous reactivity and associated U uptake behaviour compared to nZVI synthesised via the reduction of ferrous iron using sodium borohydride. Results highlight a clear difference in reactivity between the two types of CS nZVI, with greater U removal exhibited by the nanoparticles synthesised at 700 °C. The mechanism has been attributed to the CS nZVI synthesised at 700 °C exhibiting (i) a greater proportion of surface oxide Fe^2+^ to Fe^3+^ (0.34 compared to 0.28); (ii) a greater conversion of ferric citrate trihydrate [2Fe(C_6_H_5_O_7_)·H_2_O] to Fe^0^; and (iii) a larger surface area (108.67 compared to 88.61 m^2^ g^−1^).

In comparison with the borohydride-reduced nZVI, lower maximum U uptake was recorded for both types of CS nZVI. A concurrent lower decrease in solution Eh and DO was recorded for both types of CS nZVI in comparison to the borohydride-reduced nZVI, indicating that lower chemical reduction of U was achieved by the former. This is attributed to the lower mass percentage of ZVI in the CS nZVI, decreasing the chemical reduction ability of the nanoparticles. Despite this, lower U desorption in the latter stages of the experiment (>7 days) was recorded for the CS nZVI synthesised at 700 °C. The specific mechanism for this differential U uptake behaviour remains unclear, however, it is tentatively suggested that the presence of carbon black in the CS nZVI provided an addition site for U retention. Future work will seek to examine this hypothesis. Overall, it can be concluded that the borohydride-reduced nZVI were more effective than CS nZVI for U removal over relatively short timescales (e.g. <48 h), however, they were more susceptible to U desorption over extended time periods.

## References

[CR1] Abbasi WA, Streat M (1994). Adsorption of uranium from aqueous solutions using activated carbon. Sep Sci Technol.

[CR2] Cantrell KJ, Kaplan DI, Wietsma TW (1995). Zero-valent iron for the in situ remediation of selected metals in groundwater. J Hazard Mater.

[CR3] Charlet L, Liger E, Gerasimo P (1998). Decontamination of TCE- and U-rich waters by granular iron: role of sorbed Fe(II). J Environ Eng.

[CR4] Chen C, Li X, Zhao D, Tan X, Wang X (2007). Adsorption kinetic, thermodynamic and desorption studies of Th(IV) on oxidized multi-wall carbon nanotubes. Colloids Surf A.

[CR5] Choi H, Agarwal S, Al-Abed SR (2009). Adsorption and simultaneous dechlorination of PCBs on GAC/Fe/Pd: mechanistic aspects and reactive capping barrier concept. Environ Sci Technol.

[CR6] Crane RA, Noubactep C (2012). Elemental metals for environmental remediation: lessons from hydrometallurgy. Fresen Env Bull.

[CR7] Crane RA, Scott TB (2012). Nanoscale zero-valent iron: future prospects for an emerging water treatment technology. J Hazard Mater.

[CR8] Crane RA, Scott TB (2013) The effect of vacuum annealing of magnetite and zero-valent iron nanoparticles on the removal of aqueous uranium J Nanotech 2013:1–11 Article ID 173625. doi:10.1155/2013/173625

[CR9] Crane RA, Scott TB (2014). The removal of uranium onto nanoscale zero-valent iron particles in anoxic batch systems. J Nanomater.

[CR10] Crane RA, Dickinson M, Popescu IC, Scott TB (2011). Magnetite and zero-valent iron nanoparticles for the remediation of uranium contaminated environmental water. Water Res.

[CR11] Crane RA, Dickinson M, Scott TB (2015). Nanoscale zero-valent iron particles for the remediation of plutonium and uranium contaminated solutions. Chem Eng J.

[CR12] Dickinson M, Scott TB, Crane RA, Riba O, Barnes R, Hughes G (2010). The effects of vacuum annealing on the structure and surface chemistry of iron:nickel alloy nanoparticles. J Nano Res.

[CR13] Fiedor JN, Bostick WD, Jarabek RJ, Farrell J (1998). Understanding the mechanism of uranium removal from groundwater by zero-valent iron using X-ray photoelectron spectroscopy. Environ Sci Technol.

[CR14] Field TB, McCourt JL, McBryde WAE (1974). Composition and stability of iron and copper citrate complexes in aqueous solution. Can J Chem.

[CR15] Grovesnor AP, Kobe BA, Biesinger MC, McIntyre NS (2004). Investigation of multiplet splitting of Fe 2p XPS spectra and bonding in iron compounds. Surf Interface Anal.

[CR42] Gu B, Liang L, Dickey MJ, Yin X, Dai S (1998) Reductive precipitation of uranium(VI) by zero-valent iron. Environ Sci Technol 32:3366–3373

[CR16] Hoch LB, Mack EJ, Hydutsky BW, Hershman JM, Skluzacek JM, Mallouk TE (2008). Carbothermal synthesis of carbon-supported nanoscale zero-valent iron particles for the remediation of hexavalent chromium. Environ Sci Technol.

[CR17] Jabeen H, Chandra V, Jung S, Lee JW, Kim KS, Kim SB (2011). Enhanced Cr(VI) removal using iron nanoparticle decorated graphene. Nanoscale.

[CR18] Kantar C, Gillow JB, Harper-Arable R, Honeyman BD, Francis AJ (2005). Determination of stability constants of U(VI)–Fe(III)-citrate complexes. Environ Sci Technol.

[CR19] Kumar S, Loganathan VA, Gupta RB, Barnett MO (2011). An assessment of U(VI) removal from groundwater using biochar produced from hydrothermal carbonization. J Environ Manag.

[CR20] Li ZH, Jones HK, Zhang PF, Bowman RS (2007). Chromate transport through columns packed with surfactant-modified zeolite/zero valent iron pellets. Chemosphere.

[CR21] Lv X, Xu J, Jiang G, Xu X (2011). Removal of chromium(VI) from wastewater by nanoscale zero-valent iron particles supported on multiwalled carbon nanotubes. Chemosphere.

[CR22] Mellah A, Chegrouche S, Barkat M (2006). The removal of uranium(VI) from aqueous solutions onto activated carbon: kinetic and thermodynamic investigations. J Colloid Interface Sci.

[CR23] Mossman DJ, Najy B, Davis DW (1993). Hydrothermal alteration of organic matter in uranium ores, Elloit Lake, Canada: implications for selected organic-rich deposits. Geochim Cosmochim Acta.

[CR24] Nagy B, Gauthier-Lafaye F, Holliger P, Davis DW, Mossman DJ, Leventhal JS, Rigali MJ, Parnell J (1998). Organic matter and containment of uranium and fissiogenic isotopes at the Oklo natural reactors. Nature.

[CR25] O’Loughlin EJ, Kelly SD, Cook RE, Csencsits R, Kemner KM (2003). Reduction of uranium (VI) by mixed iron(II)/iron(III) hydroxide (green rust): formation of UO_2_ nanoparticles. Environ Sci Technol.

[CR26] Ponder SM, Darab JG, Mallouk TE (2000). Remediation of Cr(VI) and Pb(II) aqueous solutions using nanoscale zero-valent iron. Environ Sci Technol.

[CR27] Ragnarsdottir K, Charlet L (2000). Uranium behaviour in natural environments. Environmental mineralogy–microbial interactions, anthropogenic influences, contaminated land and waste management. Mineralog Soc Ser.

[CR28] Rahman RO, Ibrahium HA, Hung Y-T (2011). Liquid radioactive wastes treatment: a review. Water.

[CR29] Riba O, Scott TB, Ragnarsdottir KV, Allen GC (2008). Reaction mechanism of uranyl in the presence of zero-valent iron nanoparticles. Geochim Cosmochim Acta.

[CR30] Scott TB, Dickinson M, Crane RA, Riba O, Hughes G, Allen G (2010). The effects of vacuum annealing on the structure and surface chemistry of iron nanoparticles. J Nano Res.

[CR31] Scott TB, Popescu IC, Crane RA, Noubactep C (2011). Nano-scale metallic iron for the treatment of solutions containing multiple inorganic contaminants. J Hazard Mater.

[CR32] Sheng G, Shao X, Li Y, Li J, Dong H, Cheng W, Gao X, Huang Y (2014). Enhanced removal of uranium(VI) by nanoscale zerovalent iron supported on Na–bentonite and an investigation of mechanism. J Phys Chem A.

[CR33] Shin Y, Bae S, Lee W (2013). Formation of surface mediated iron colloids during U(VI) and nZVI interaction. Adv Environ Res.

[CR34] Smith RM, Martell AE (1993). NIST Critical Stability constants of metal complexes database; NIST Standard Reference Database 46.

[CR35] Suna Y, Ding C, Cheng W, Wang X (2014). Simultaneous adsorption and reduction of U(VI) on reduced graphene oxide-supported nanoscale zerovalent iron. J Hazard Mater.

[CR36] Üzüm C, Shahwan T, Eroğlu AE, Hallam KR, Scott TB, Lieberwirth I (2009). Synthesis and characterization of kaolinite-supported zero-valent iron nanoparticles and their application for the removal of aqueous Cu^2+^ and Co^2+^ ions. Appl Clay Sci.

[CR37] Wang CB, Zhang WX (1997). Synthesizing nanoscale iron particles for rapid and complete dechlorination of TCE and PCBs. Environ Sci Technol.

[CR38] Wang Y-Q, Zhang Z-B, Liu Y-H, Cao X-H, Liu Y-T, Li Q (2012). Adsorption of U(VI) from aqueous solution by the carboxyl-mesoporous carbon. Chem Eng J.

[CR39] Yan S, Hua B, Bao ZY, Yang J, Liu CX, Deng BL (2010). Uranium (VI) removal by nanoscale zerovalent iron in anoxic batch systems. Environ Sci Technol.

[CR40] Yan S, Chen Y, Xiang W, Bao Z, Liu C, Deng B (2014). Uranium(VI) reduction by nanoscale zero-valent iron in anoxic batch systems: the role of Fe(II) and Fe(III). Chemosphere.

[CR41] Zhang W-x (2003). Nanoscale iron particles for environmental remediation: an overview. J Nano Res.

